# Metformin and acupuncture for polycystic ovary syndrome: A protocol for a systematic review and meta-analysis

**DOI:** 10.1097/MD.0000000000019683

**Published:** 2020-04-03

**Authors:** Yang Gao, Suyun Xu, Yifeng Shen, Tingting Liao, Shiruo Hu, Shan Zhou, Qiu Chen

**Affiliations:** Hospital of Chengdu University of Traditional Chinese Medicine, PR China.

**Keywords:** acupuncture, metformin, polycystic ovary syndrome, protocol, systematic review and meta-analysis

## Abstract

**Backgrounds:**

Polycystic ovary syndrome (PCOS) is a common endocrine disorder in women and can lead to serious social burdens associated with various reproductive and metabolic abnormalities. Studies have demonstrated that metformin can reduce liver glucose in PCOS, lower testosterone levels and increase peripheral insulin sensitivity. There has been also evidence suggesting acupuncture may influence ovulation (release of the egg) by affecting levels of various hormones. We will conduct a systematic review and meta-analysis is to compare the efficacy and safety of metformin with or without acupuncture in PCOS.

**Methods and analysis:**

We will search publications from Web of Science, PubMed, Science Direct, Wan Fang Data Knowledge Service Platform, Chinese Biomedical Literature Database (CBM), Chinese Scientific Journal Database (VIP database), China National Knowledge Infrastructure (CNKI) and EMBASE, which should be published from inception to February 2020. Two researchers will independently perform the selection of the studies, data extraction, and synthesis. The Cochrane Risk of Bias Tool will be used to evaluate the risk of bias of the randomized controlled trials. Statistical analysis will be performed by using the Cochrane Review Manager (RevMan 5.3) software. The *I*^2^ test will be used to identify the extent of heterogeneity. We will use the Egger funnel chart to evaluate possible publication biases, in addition, when possible we will perform a subgroup/meta-regression analysis. The strength of the evidence will be assessed according to the Grading of Recommendations Assessment, Development, and Evaluation (GRADE).

**Results and conclusion:**

This study will systematically evaluate the efficacy and safety of Metformin combined with acupuncture in the treatment of PCOS, thus providing evidence to the clinical application of this combination therapy. The results will be published in a peer-reviewed journal.

## 1 Introduction

Polycystic ovary syndrome (PCOS) is one of the most common heterogeneous endocrine disorders that is characterized by the clinical signs of oligo-amenorrhea (infrequent or very light menstruation), infertility (failure to conceive) and hirsutism (excessive hair growth).^[1,2]^ PCOS has become a major healthcare issue that deserves an increase in focus, from both clinical and public health perspectives. The prevalence of PCOS ranges from 6% to 20% depending on definitions and populations studied.^[3,4]^

Insulin resistance (IR) plays a key role in the pathophysiology of PCOS and IR affects 85% (75% of lean and 95% of overweight) of cases. This condition is associated with women's reproductive abnormalities, including fetal macrosomia, polyhydramnios, operative delivery, high perinatal mortality, and neonatal metabolic complications.^[5,6]^ In addition, The prevalence of IR in women with PCOS is likely to be exacerbated by obesity. Weight loss reduced testosterone and IR, although there was insufficient evidence to determine whether reproductive outcomes were improved.^[7]^ Lifestyle management is the first-line therapy in PCOS for the prevention of weight gain and for weight loss.^[8]^ However, as in the general population, the efficacy of lifestyle management for established obesity has been limited in PCOS,^[7]^ with new approaches needed. Metformin has been commonly used to increase insulin sensitivity in women with PCOS. The predicted and confirmed benefits of metformin include decreased hepatic glucose, decreased testosterone level, and high peripheral insulin sensitivity.^[9]^ However, the exact role of metformin in the management of women with PCOS is quite controversial. It often suffers from intrinsic shortcomings such as adverse reactions, weak weight loss benefits and uncertain reproductive effects.^[10]^ Acupuncture involves the insertion of needles into specific anatomical points (termed acupoints) and has been used in eastern Asian countries for thousands of years. Recently, the use of acupuncture in reproductive endocrinology and infertility has gained increased popularity worldwide.^[11]^ Several clinical and animal experimental studies indicate that acupuncture is beneficial for ovulatory dysfunction in PCOS.^[12,13]^ Acupuncture has also been reported to potentially improve insulin sensitivity and to decrease testosterone in patients with PCOS.^[14,15]^

A number of systematic reviews have been conducted on the efficacy of metformin or acupuncture on outcomes in PCOS including weight loss, ovulation, menstruation, hyperandrogenism, and IR.^[16–19]^ However, there is currently no systematic review comparing the effect of metformin and acupuncture in PCOS. The primary aim of this study is to undertake a comprehensive systematic review and meta-analysis to compare the efficacy and safety of metformin with acupuncture or single used in PCOS.

## 2 Methods and analysis

This protocol is conducted according to the Preferred Reporting Items for Systematic Reviews and Meta-Analysis Protocol (PRISMA-P) statement guidelines and the Cochrane Handbook for Systematic Reviews of Interventions. This systematic review protocol has been registered on OSF as 10.17605/OSF.IO/68WQ5.

### 2.1 Inclusion and exclusion criteria

#### 2.1.1 Types of studies

This study contains only human randomized controlled trials, without any restrictions on language, date of transmission or type of publication. This systematic review will exclude non-randomized controlled trials, quasi-randomized controlled trials, retrospective studies, retrospective studies, case reports, non-controlled trials, and animal mechanism studies. For included trials, investigators need to accurately report randomized methods, diagnostic criteria, details of interventions, and assessment of efficacy. The duration of treatment and follow-up of patients is unlimited.

#### 2.1.2 Types of participants

Standard diagnosis of PCOS patients will be included in the analysis, regardless of their age, ethnicity, and background, according to the European Society of Human Reproduction and Embryology (ESHRE) and the American Society of Reproductive Medicine (ASRM) consensus in Rotterdam.^[20]^

#### 2.1.3 Types of interventions

##### 2.1.3.1 Experimental intervention

We define metformin +acupuncture (both manual and electrical stimulation) as the experimental intervention. Metformin included in this study will not be limited by dose or dosage form. This review will comprise clinical trials that focus on acupuncture with the insertion of needles into selected acupoints up to definite therapeutic depths regardless of the site or type of treatment, needling techniques and stimulation method. Besides, there will be no restrictions in terms of the needle materials, frequency of treatment sessions, and treatment courses. At the same time, other acupuncture treatments such as auricular acupuncture, fire needling, intradermal needling, 3-edged needling, plum blossom needling, warm needling, laser acupuncture, point injection, pricking blood, moxibustion, pharmaco acupuncture, transcutaneous electrical nerve stimulation, cupping and acupressure will be excluded.

##### 2.1.3.2 Comparison interventions

Comparison interventions will be either metformin alone or metformin combined with placebo acupuncture. Sham acupuncture is also known as placebo acupuncture and uses techniques that are not intended to stimulate known acupuncture points.

#### 2.1.4 Types of outcome measures

Primary outcomes will include total response rate, reproductive [menstruation, pregnancy rate, total testosterone, SHBG, free androgen index (FAI), dehydroepiandrosterone sulphate, hirsutism, acne], BMI, ovulation rate and pregnancy rate.

Secondary outcomes will consider anthropometric indicators [waist circumference, waist to hip ratio (WHR), subcutaneous and visceral fat], metabolic surrogate markers of IR (fasting insulin, oral glucose tolerance test (OGTT) insulin, insulin area under the curve (AUC), the homeostatic model assessment (HOMA) index, insulinogenic index, insulin sensitivity index, quantitative insulin sensitivity check index (QUICKI)) and glucose intolerance (impaired fasting glucose (IFG), IGT or DM2), highly sensitive C reactive protein (hsCRP), lipids], and psychological [quality of life] parameters.

### 2.2 Data sources and search strategy

The following databases will be searched from their inception to February 2020 for relevant studies: Web of Science, PubMed, Science Direct, Wan Fang Data Knowledge Service Platform, Chinese Biomedical Literature Database (CBM), Chinese Scientific Journal Database (VIP database), China National Knowledge Infrastructure (CNKI) and EMBASE. Two reviewers (SH and SZ) will independently search the studies. Any differences will be resolved through discussion with a third author (GY). We will also search the US National Institutes of Health Ongoing Trials Register, the WHO International Clinical Trials Registry Platform, Chinese Clinical Trial Registry and Google Scholar for any relevant ongoing or unpublished trials. For a comprehensive search, a search strategy that combines MeSH terms and free words will be adopted by us. Table 1 shows that the search strategy for PubMed and modified search strategies will be applied to other databases.

**Table 1:**
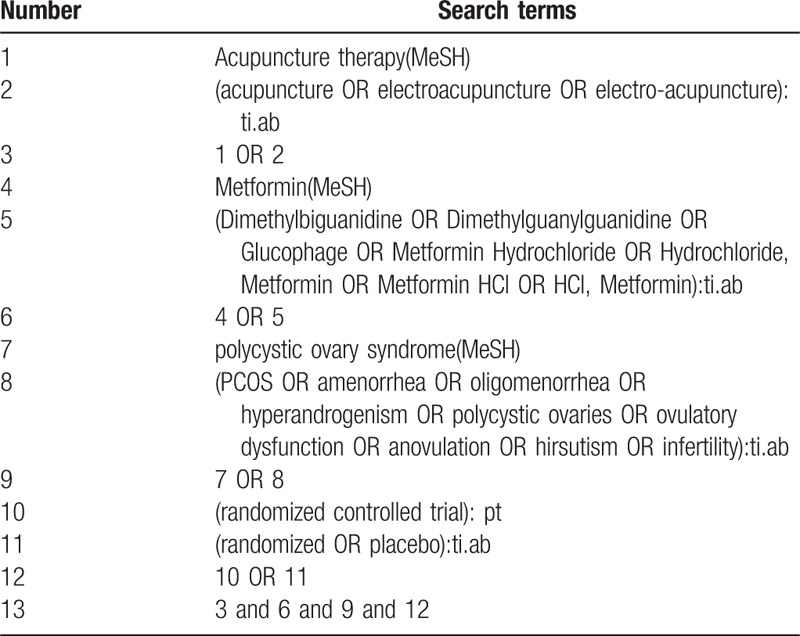
Search strategy for PubMed.

### 2.3 Data collection and analysis

#### 2.3.1 Study selection

**Figure 1:**
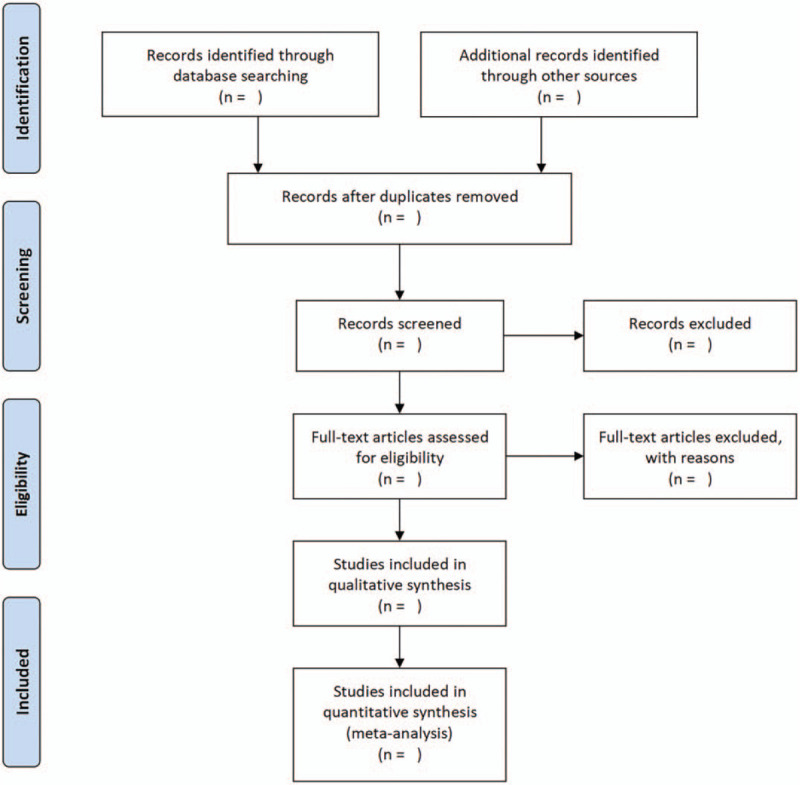
Flow diagram of study selection process.

Two researchers will independently assess all relevant studies and select eligible articles that meet inclusion criteria by reviewing the titles and abstracts. The full-texts of articles will be examined for further evaluation. In case of a discrepancy between review authors, an agreement will be made by the discussion with the corresponding author (GY). The procedure of study selection will be summarized by using a Preferred Reporting Items for Systematic Reviews and Meta-Analyses protocols flow diagram (Fig. 1).

#### 2.3.2 Data extraction and management

Before data extraction, a standard data extraction form (Excel) containing specified outcomes will be created according to the inclusion. Two reviewers (YS and TL) will then extract data independently such as the first author, year of publication, diagnostic information, course of the disease, sample size, age, intervention details, control and outcome, treatment time, follow-up time, and adverse events. Any disagreements will be resolved through discussion or consultation between the 2 reviewers if necessary. Final determination from a third reviewer (QC) will be sought. When the data of articles are insufficient or ambiguous, one of the authors will in contact with the original author to request detailed about the research by e-mail or telephone or estimating the data.

#### 2.3.3 Risk of bias assessment

To assess the methodological quality of the included studies, 2 reviewers (YS and SX) will independently use the Cochrane risk of bias tool to examine 7 aspects: random sequence generation, allocation concealment, blinding of participants and personnel, blinding of outcome assessment, incomplete data assessment, selective outcome reporting, and other sources of bias.

#### 2.3.4 Measures of treatment effect

We will conduct a meta-analysis if the studies can be combined. The risk ratio (RR) with 95% confidence intervals (CI) will be calculated for dichotomous data. Standardized mean difference (SMD) with 95% CI will be calculated for continuous data. We will provide a narrative synthesis of the outcomes and results of the studies if a meta-analysis is not possible.

#### 2.3.5 Unit of analysis issue

Only the first experimental period date will be considered in randomized cross-over trials. Studies with multiple intervention groups, this review will combine experimental and control intervention groups into a single respectively group to avoid a unit of analysis issue.

#### 2.3.6 Assessment of heterogeneity

According to the guidelines of the Cochrane Handbook for Systematic Reviews of Interventions, we will choose the *I*^2^ statistic and the chi-square test with a significance level of *P* < .1 to measure heterogeneity among the studies in every analysis. when the *I*^2^ value is less than 50%, the study will not be considered to have statistical heterogeneity. While the *I*^2^ value exceeds 50%, significant statistic heterogeneity exists among the trial and meta-analysis will not be performed. And subgroup analysis will be conducted to determine the reason.

#### 2.3.7 Assessment of reporting bias

If our review has a sufficient number of included trials that are available in the meta-analysis, a funnel plot and statistic test will be generated to analyze the potential reporting bias as well as small study effects.

#### 2.3.8 Data synthesis and analysis

When *I*^2^ < 50% is regarded as no evidence of substantial statistical heterogeneity, a fixed-effects model will be used for pooled data. When *I*^2^ ≥ 50% is regarded as substantial statistical heterogeneity, a random-effects model will be adopted to synthesize the data and reach a conclusion more cautiously. If the data is not suitable for combining quantitative synthesis, in this case, a systematic narrative description will be provided with the information presented in the text to summarize and explain the characteristics and findings of the individual studies.

#### 2.3.9 Subgroup analysis

To identify substantial heterogeneity, subgroup analysis will be implemented according to characteristics of patients, type of intervention and outcome measures.

#### 2.3.10 Sensitivity analysis

When studies are adequate, sensitivity analysis will be adopted for primary outcomes to explore the robustness of conclusions if feasible, and assess the impact of methodological quality, sample size and missing data. Sensitivity analysis will be conducted by removing lower quality studies if heterogeneity remains after subgroup analysis or studies with incomplete results according to the STRICTA checklist. The meta-analysis will be carried out again after trials of lower quality have been excluded. The results of these meta-analyses then will be compared and discussed according to their sample size, the strength of evidence and influence on the pooled effect size. However, if all included studies have a high risk of bias, we will not carry out sensitivity analyses.

#### 2.3.11 Grading the quality of evidence

The GRADE system will be used for evaluating the quality of evidence in systematic reviews. The evaluation included bias risk; heterogeneity; indirectness; imprecision; publication bias. And each level of evidence will be made “very low,” low,” “moderate,” or “high” judgment.

## 3 Discussion

PCOS is a highly prevalent problem. Metformin as drug treatment is a conventional approach. In recent years, acupuncture has been widely used in PCOS. The purpose of this systematic analysis is to assess the efficacy and safety of the addition of acupuncture to metformin to explore the application of the combination therapy on patients experiencing PCOS. It is a potential therapy for PCOS, and several relevant researches have emerged. This systematic review will provide current evidence on the effectiveness and safety of metformin combined with acupuncture for PCOS. These findings may help provide guidance to clinicians in the treatment of PCOS. The results of this report will be disseminated after peer review and publication.

## Author contributions

**Conceptualization:** Yang Gao, Suyun Xu, Qiu Chen.

**Data curation:** Yifeng Shen, Tingting Liao.

**Formal analysis:** Yang Gao, Suyun Xu.

**Funding acquisition:** Qiu Chen.

**Investigation:** Shiruo Hu, Shan Zhou.

**Methodology:** Yang Gao, Suyun Xu, Qiu Chen.

**Software:** Suyun Xu.

**Supervision:** Qiu Chen.

**Writing – original draft:** Yang Gao.

**Writing – review & editing:** Suyun Xu, Yifeng Shen, Qiu Chen.
